# Altered T-cell repertoire diversity in septic shock patients

**DOI:** 10.1186/cc13413

**Published:** 2014-03-17

**Authors:** N Takeyama, A Tomino, M Hashiba, A Hirakawa, T Hattori, H Miyabe

**Affiliations:** 1 Fujita Health University, Aichi, Japan; 2 Fuita Health University, Aichi, Japan

## Introduction

The occurrence of sepsis-induced immune suppression is associated with multiple organ dysfunctions, although the exact role of T-cell malfunction is obscure. We investigated the impact of sepsis on the adaptive immune system and to monitor T-cell receptor (TCR) diversity.

## Methods

TCR diversity was analyzed in peripheral blood mononuclear cells (PBMCs) isolated from septic shock patients at three time points (days 1, 3 and 7 after diagnosis of septic shock). TCR diversity was measured in genomic DNA isolated from PBMCs using the Human Immun TraCkeRb test (ImmunID Technologies, Grenoble, France)[1]. Multi-N-plex PCR was performed using a primer specific to a V gene family and several primers specific to J segments. The signal is measured as a function of the fluorescence intensity of the reference marker. Rearrangement validation and map generation were detected and analyzed using the Constel'ID software (ImmunID Technologies). HLA-DR expression on CD14 cells was measured by flow cytometry.

## Results

TCR diversity was markedly decreased in septic patients at day 1 compared with healthy volunteers. A recovery of TCR diversity was observed at days 3 and 7 except for dead patients. HLA-DR expression was significantly decreased in septic patients at day 1. The total lymphocyte count reduced in septic patients at day 1, but the lymphocyte count recovered at days 3 and 7 except for dead patients.

## Conclusion

We observed a lower TCR diversity and lymphopenia in the early stages of septic shock. A quick recovery of tCr diversity and lymphocyte count was observed in live patients. On the other hand, dead patients stayed on the lower level over the course of the study. These results suggest that altered TCR diversity and lymphopenia might participate in increased mortality and susceptibility to secondary nosocomial infections.
